# A novel approach of recombinant laterosporulin production using the N-SH2 domain of SHP-2

**DOI:** 10.1186/s12896-021-00721-7

**Published:** 2021-10-21

**Authors:** Simin Salehzadeh, Mohammad Tabatabaei, Abdollah Derakhshandeh, Hamidreza Karbalaei-Heidari, Nasrin Kazemipour

**Affiliations:** 1 Department of Pathobiology, School of Veterinary Medicine, Shiraz University, Shiraz, Iran; 2 Department of Biology, Faculty of Science, Shiraz University, Shiraz, Iran; 3 Department of Basic Science, School of Veterinary Medicine, Shiraz University, Shiraz, Iran

**Keywords:** N-SH2 domain of SHP-2, Protein, Laterosporulin, Disulfide bond

## Abstract

**Background:**

The current study was aimed at evaluating the role of the N-SH2 domain of SHP-2 as a partner protein in the expression of a toxic peptide, laterosporulin (LTS). We also investigated its effects on the formation of the disulfide bond and functional folding of the peptide in vitro. The N-SH2-LTS protein was expressed as a His-tagged fusion protein, capable of undergoing enzymatic cleavage.

**Results:**

Based on the data presented herein, the total yield of the folded fusion protein from inclusion bodies was found to be about 105 mg/l, demonstrating a high-level of heterologous expression. After enzymatic cleavage, 1.5 mg of the folded recombinant laterosporulin was obtained from each 10 mg of the fusion protein. The purity of the recombinant laterosporulin was analyzed by RP-HPLC, to yield peptides with suitable purity (85%).

**Conclusions:**

Our findings indicated the advantages of using the N-SH2 domain of SHP-2 as a rapid and easy approach not only in producing easy target proteins but also in its function as a chaperone. N-SH2 domain of SHP-2 can influence on the purification of laterosporulin at reasonable yield and in a cost-effective fashion. The N-SH2 domain of SHP-2 as a protein chaperone may be potentially favorable to produce other proteins with disulfide bonds.

**Supplementary Information:**

The online version contains supplementary material available at 10.1186/s12896-021-00721-7.

## Background

Regarding the recent increase in the number of antibiotic-resistant pathogens, scientific efforts now focus more on alternative candidates [1, 2]. Bacteriocins, low-molecular-weight antimicrobial peptides (AMPs), are known to be natural peptide antibiotics, ribosomally synthesized by various bacteria and released extracellularly. They perpetrate bacteriostatic or bactericidal activity towards other bacteria [3, 4]. Bacteriocins can be a very promising alternative for antibiotics due to their remarkable potency, narrow and broad killing spectrums, favorable stability, and low toxicity. These unique properties can be effectively applied to clinical practices [4–6].

The ultimate production method of these active peptides in large amounts is by recombinant production. This entails the genetic manipulation and production of recombinant plasmids encompassing genes that encode the corresponding peptides [7].

In 2012, a novel bacteriocin produced by *Brevibacillus* sp. strain GI-9 was introduced and purified to evaluate its antibacterial spectrum called laterosporulin [8]. This heat‐stable peptide with 50 amino acids in length and a molecular weight of 5750 Da, is a cysteine‐rich peptide consisting of three disulfide bonds in its structure [8]. Based on the available data, laterosporulin exhibited four β-strands forming a twisted β-sheet with three intramolecular disulfide bonds*,* stabilizing its structure. Laterosporulin presents structural homology and sequence identity to numerous human defensins and has been introduced as an antibacterial agent to target specifically non-multiplying bacteria [9]. This peptide is generally more resistant to heat and pH change and no changes in its anti-microbial activity have been observed when exposed to proteolytic enzymes [8]. As previously indicated, the inhibitory effect of this peptide on the growth of bacteria, such as *Listeria*, *Pseudomonas* and *B. cereus*, has been observed. Laterosporulin exhibits a broad antibacterial effect on *Staphylococcus aureus* and *Bacillus subtilis*. [8].

The existence of disulfide bonds in the peptide structure may be a problem in the recombinant production of these types of peptides. This may result in the production of insoluble or inactive peptides due to incorrect folding when these peptides are overexpressed in *Escherichia coli* [10]. To produce recombinant bacteriocin, various carrier proteins are applied for designing desired plasmids and consequently fusion expression of antimicrobial peptides [11].

Fairlie et al. validated that the engineered mutant of the N-SH2 domain of SHP-2 as a fusion protein could improve the expression of short peptides with one disulfide bond [12]. Several activities of the Src homology 2 (SH2) domain have been previously described in different kinds of proteins in a wide variety of eukaryotes and metazoans. These analyses incorporated transcription factors, molecular adaptors, and tyrosine kinases [13, 14].

The absence of a cysteine residue in the N-SH2 domain of SHP-2 [12] was a principal feature for selecting this fragment as a fusion protein in this research. This was advantageous in preventing a disulfide bond formation between the partner and laterosporulin peptide.

After the application of bioinformatics to evaluate the structure of the N-SH2 domain and its possible positive effects on the expression of recombinant laterosporulin, the N-SH2 domain as a carrier protein was used to produce recombinant fusion bacteriocin for the first time. The possible effects of the N-SH2 domain on the production of a soluble form, expression, and folding of laterosporulin were investigated.

To investigate the role of the N-SH2 domain as a carrier protein, a recombinant pET-22b(+) plasmid comprising an N-SH2 domain and a laterosporulin gene fragment, was constructed and overexpressed in *E. coli*. The N-SH2-LTS fusion protein was subsequently purified and the partner was removed. After that, its antibacterial effect on *S. aureus* was evaluated.

## Results

### Bioinformatics analysis

**Fig. 1:**
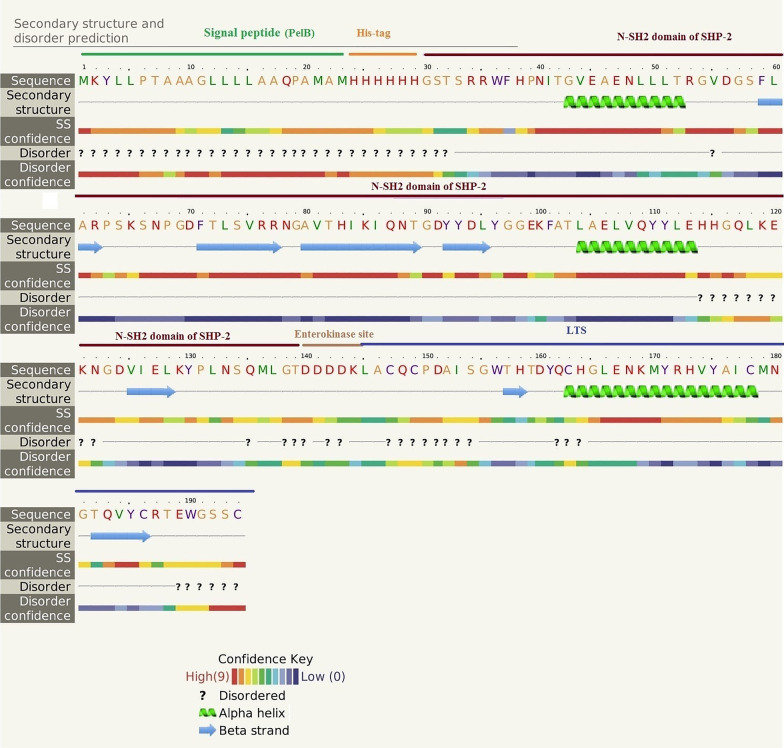
The secondary structure of the N-SH2-LTS fusion protein was predicted by Phyre2 server

The predicted secondary structure of the N-SH2-LTS fusion protein is shown in Fig. 1. Analysis of solubility prediction of the N-SH2-LTS fusion protein showed solubility of 35.4% as compared to the average solubility of *E. coli* soluble proteins (40%). The mean solubility prediction of soluble proteins (*E. coli*) was obtained from experimental data [15]. Based on the results, the structure of the fusion protein after modeling did not show very significant changes in terms of solubility compared to the natural structure of laterosporulin (36.3%).

**Fig. 2:**
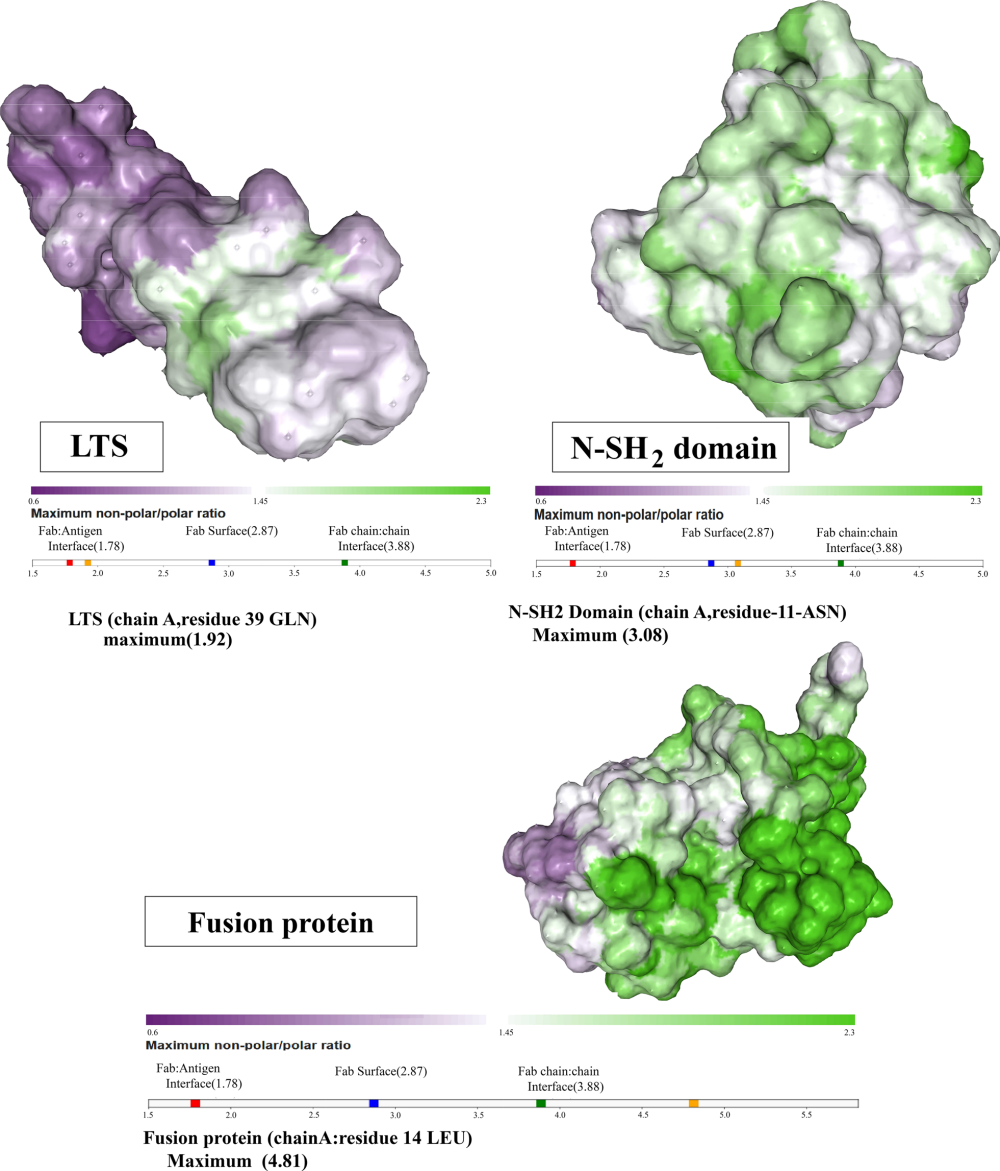
Surface patches of LTS, N-SH2 domain and Fusion protein. Regions is highlighted from purple region with low NPP ratio to green with high NPP ratio (hydrophobic patch)

Protein-sol patches software calculated hydrophobic parts on the protein surface based on the PDB format (Fig. 2), and identified areas that may affect the behavior and stability of the protein structure. The software depicted the ratio of polar and non-polar regions on the laterosporulin, N-SH2 domain, and the fusion protein. The hydrophobic areas are revealed in green as depicted in the Fig. 2. The N-SH2 domain is more hydrophobic than laterosporulin. However, its fusion with the laterosporulin peptide resulted in an increased hydrophobicity of the structure.

### Gene cloning and recombinant fusion protein expression

**Fig. 3:**
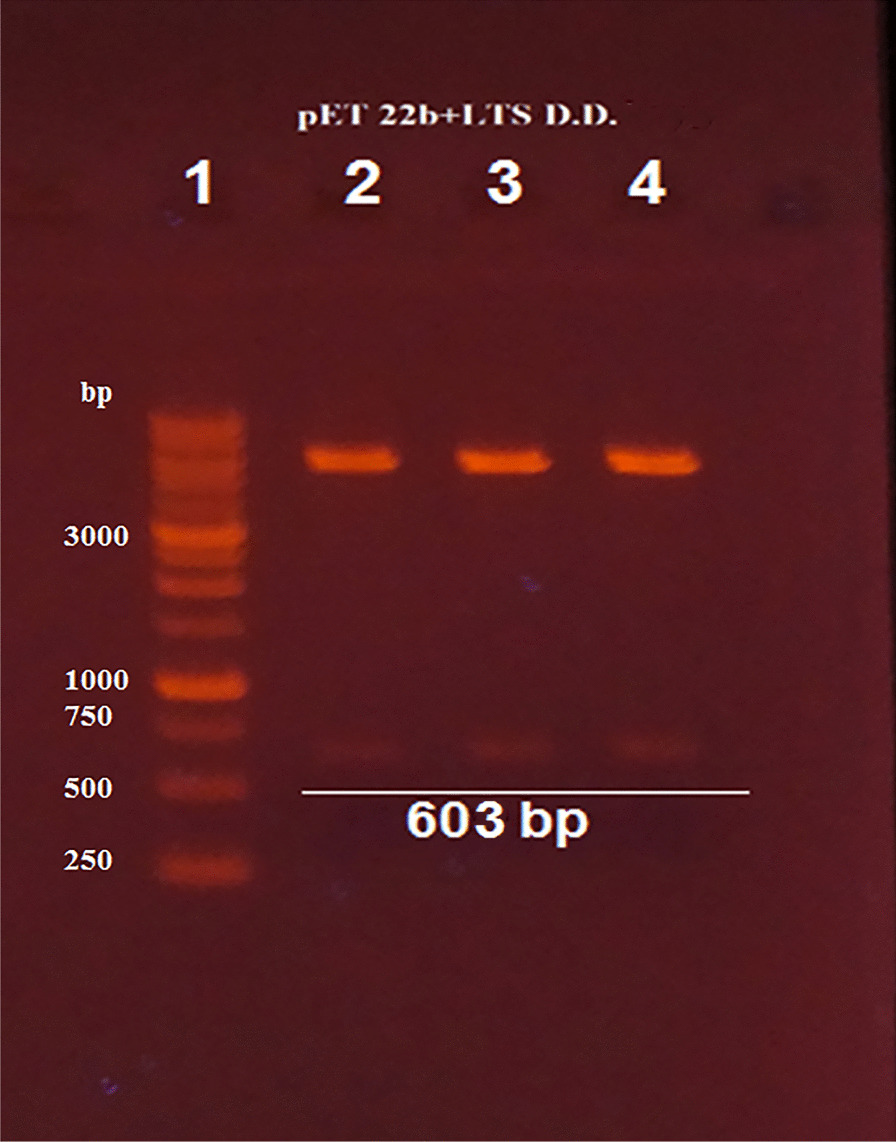
Digestion of recombinant plasmid pET-22b(+) containing 603 bp fragment by the restriction enzymes digestion (*Xho*I and *Nde*I). (Lane 1): 1 Kb DNA Ladder RTU, (Lanes 2, 3 and 4): digestion of recombinant plasmids pET-22b(+) containing 603 bp fragment (pET-22b-N-SH2-LTS1, pET-22b-N-SH2-LTS2, pET-22b-N-SH2-LTS3) by the restriction enzymes (*Xho*I and *Nde*I)

Recombinant plasmid named pET-22b-N-SH2-LTS was constructed by cloning of synthetic fragment of 603 bp (carrying PelB, N-SH2 domain, enterokinase cleavage site sequence and LTS) into pET-22b(+) plasmid predigested with *Nde*I and *Xho*I restriction enzymes. The success of the cloning was checked by double RE *Nde*I and *Xho*I digestion (Fig. 3) and confirmed by DNA sequencing.

**Fig. 4:**
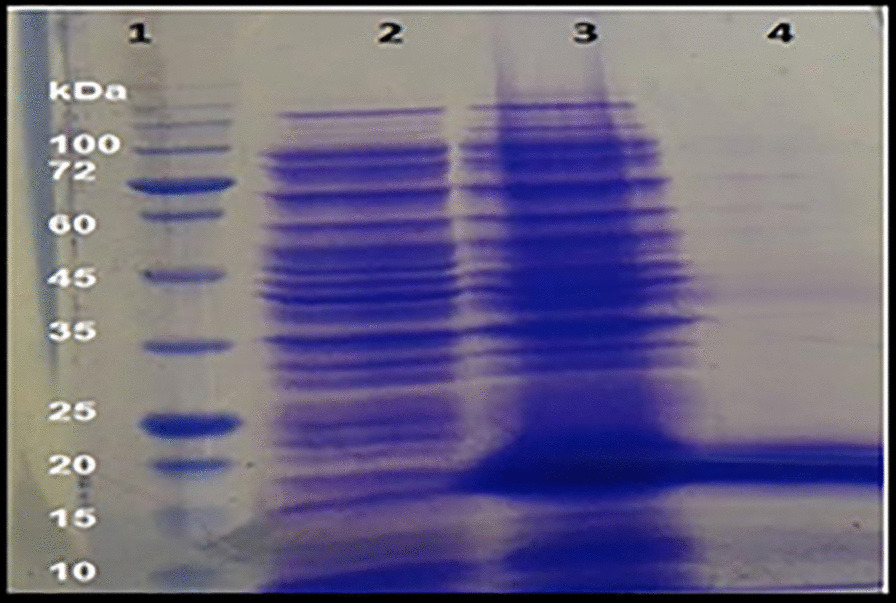
The presence of a strong expressed protein band was visualized on SDS-gel with a molecular weight of 21.78 kDa in cell lysate. (Lane 1): Protein marker, (Lane 2): Transformed *E. coli* cell lysates before induction with IPTG, (Lane 3): Transformed *E. coli* cell lysates after 18 h induction with 0.5 mM IPTG, where the presence of a strong expressive protein is shown at a weight of 21.78 kDa, (Lane 4): Purified denatured fusion protein. Please note that the uncropped original SDS-gel image is shown in Additional file 1: Fig. S1

**Fig. 5:**
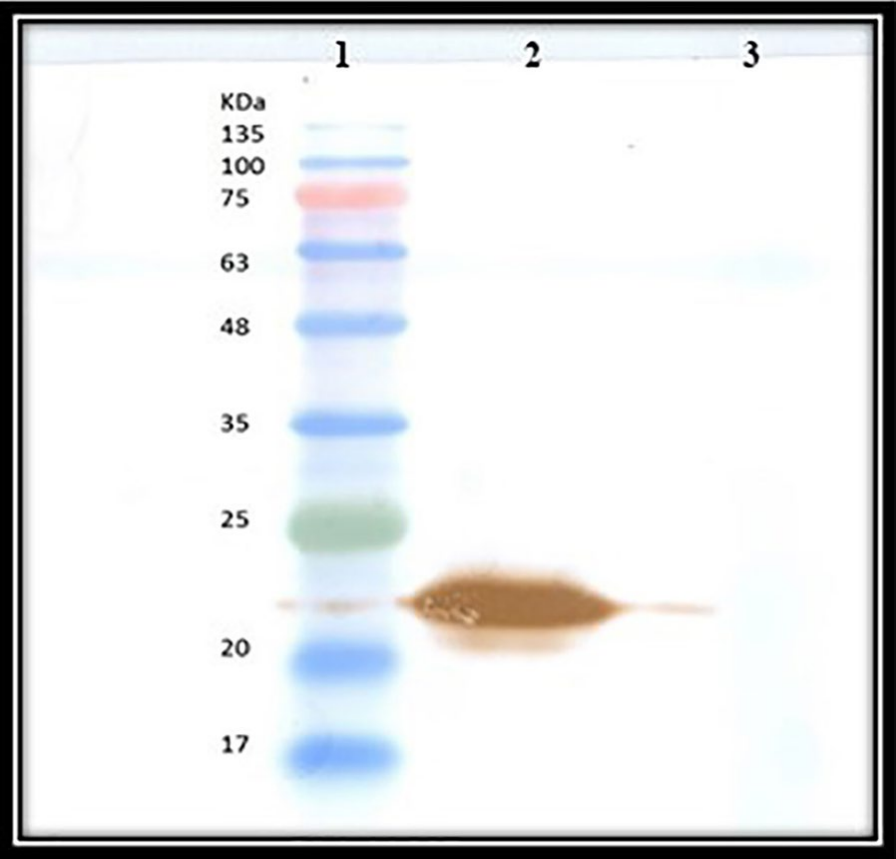
Western blot analysis of *E. coli* cells lysates in order to confirm the molecular weight of expressed fusion protein. (Lane 1): Protein ladder, (Lane 2): Fusion protein was visualized at a weight of 21.78 kDa, (Lane 3): cells lysates without induction with IPTG. Please note that the uncropped original image is shown in Additional file 1: Fig. S2

After transforming the pET-22b-N-SH2-LTS to *E. coli* BL21 cells, 0.5 mM IPTG was used to induce the expression of the fusion protein. The presence of a strong expressive protein was visualized at a weight of 21.78 kDa in the cell lysate (Fig. 4, Lane 3). Western blotting with anti His antibody also confirmed the molecular weight of the expressed fusion protein at position of 21.78 kDa (Fig. 5).

### Purification and in vitro refolding of fusion protein

Fusion protein was loaded on a Ni-agarose resin column under denaturing conditions. Then, the purified fusion protein was eluted by applying the buffer containing 250 mM imidazole (Fig. 4, Lane 4). The total yield of the denatured protein was about 134.75 mg/l, indicating a high level of fusion protein expression. The denatured fusion protein was heavily sensitive to the high ionic strength of the dialysis buffer (20 mM Tris–HCl; 300 mM NaCl, pH 8.0). It quickly precipitated in the dialysis bag as a result, when taking urea out from the buffer containing the protein. The denatured fusion protein was then refolded by dialysis against buffer containing 20 mM Tris–HCl, pH 8.0 at 4°C for 24 h. The final yield of the re-folded soluble protein was around 80% (105 mg/l).

### Enzymatic cleavage and purification of recombinant laterosporulin

**Fig. 6:**
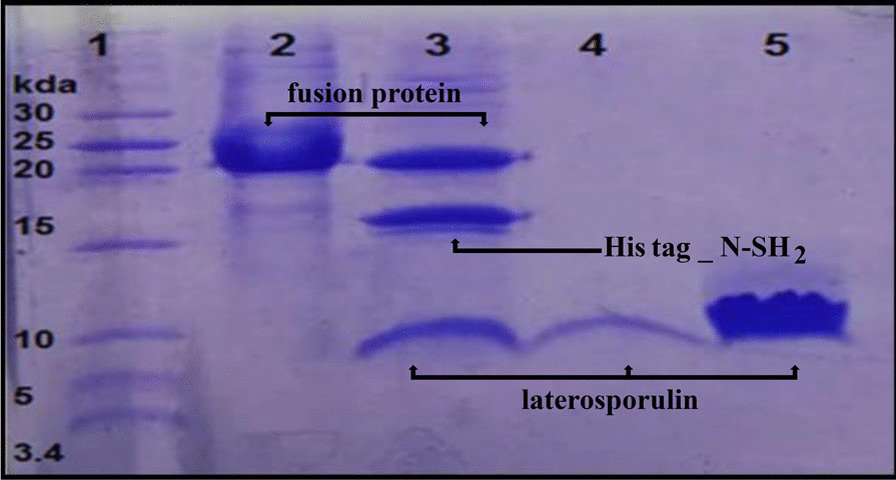
Tris-Tricine SDS-PAGE (15% polyacrylamide gel) profiles of fusion laterosporulin before and after enterokinase digestion. (Lane 1): Protein ladder, (Lane 2): Undigested refolded fusion protein at a weight of 21.78 kDa, (Lane 3): Digested fusion protein with enterokinase, (Lane 4): The purified recombinant laterosporulin (supernatant) at a weight of 5.75 kDa, (Lane 5): Highly concentrated laterosporulin. Please note that the uncropped original tris-tricine SDS-PAGE gel image is shown in Additional file 1: Fig. S3

The refolded fusion protein was sensitive to the high ionic strength of the buffer and it precipitated over time. However, this sensitivity declined when 50 mM NaCl was present. Since in the absence of the salt the efficiency of the cleavage enzyme was very low for removing fusion tags, 50 mM NaCl was added to the buffer inevitably. During the time-dependent reaction, the digested N-SH2 partner along with the undigested fusion protein precipitated leaving the purified recombinant laterosporulin in the supernatant. After the completion of the digestion reaction, 1.5 mg of the recombinant laterosporulin was obtained from 10 mg of the initial fusion protein. Since the ratio of molecular weight of laterosporulin to fusion protein was 1:4, the yield of this reaction was calculated as 59.7%, indicating a good efficiency. The results of the digestion process and the presence of laterosporulin peptide are shown in Fig. 6.

**Fig. 7:**
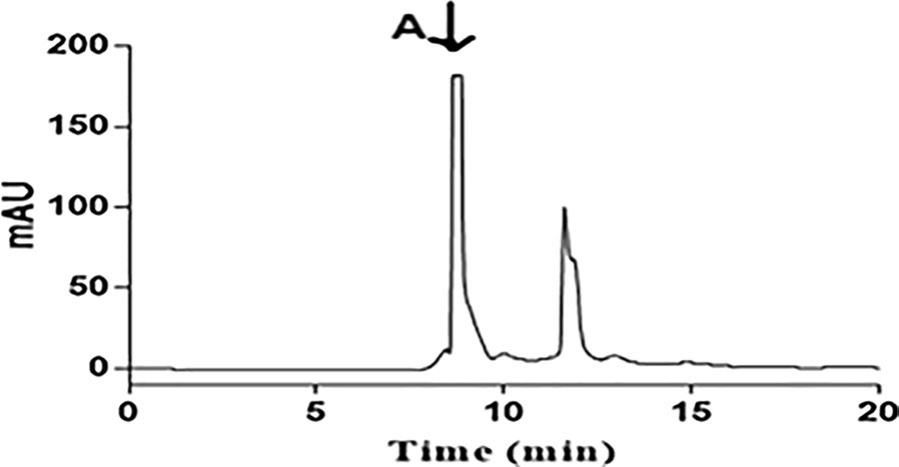
RP-HPLC chromatogram of recombinant laterosporulin. The arrow (A) corresponds to the peak of laterosporulin eluted from a TSK reverse phase C_18_ column

The purity of the recombinant laterosporulin was analyzed by Tris-Tricine SDS-PAGE (Fig. 6) and RP-HPLC (Fig. 7). On Tris-Tricine SDS-PAGE it is possible to see a strong pure band corresponding to 5.75 kDa molecular weight of purified laterosporulin (Fig. 6, Lanes 4 and 5). Hence, the major peak in HPLC chromatogram (retention time of 8 to 10 min) is that of the laterosporulin peptide, with a purity of about 85%. Probably, the second peak in RP-HPLC corresponds to other forms of laterosporulin peptide that have different levels of hydrophobicity and folding type (Fig. 7).

### Evidence of similar structural characteristics

**Fig. 8:**
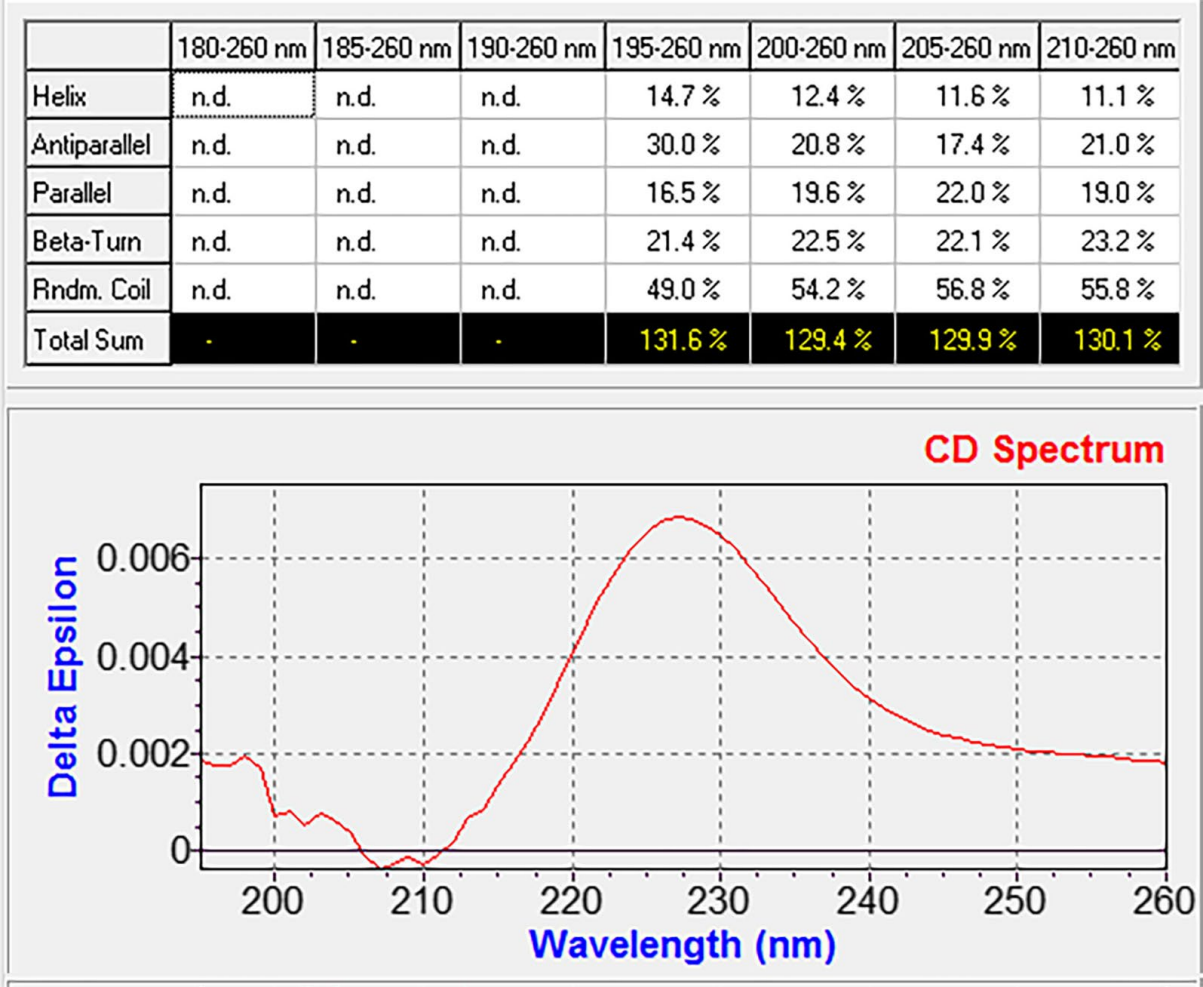
Experimental Far-UV CD spectra of laterosporulin. Far-UV CD data showed that more than 50% of the predicted structure was related to β structures. The presence of a positive band at 228 nm can be a reason for the formation of disulfide bonds [9]

The Far-UV CD was applied to evaluate the secondary structure of the recombinant laterosporulin peptide. An atypical CD spectrum was observed for the recombinant laterosporulin, with a positive band at 228 nm. The CD spectrum analysis showed the secondary structure of peptide (Fig. 8). Furthermore, the CD spectrum of the recombinant laterosporulin in the range of 195–260 nm by CDNN software showed that more than 50% of the predicted structure was related to β structures.

### Antimicrobial test

**Fig. 9:**
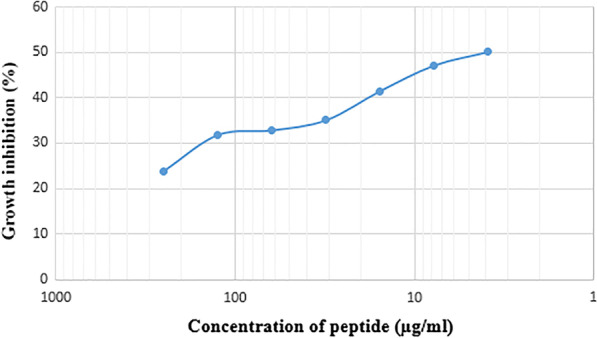
Relationship between the percentage of growth inhibition and the concentration of recombinant laterosporulin in the antibacterial test. By decreasing the concentration of peptide, an increase in growth inhibition was observed

After 24 h incubation of the recombinant bacteriocin with *S*. *aureus*, the OD of samples was recorded by an ELISA Reader. These findings were compared to the control samples containing an indicator bacterial culture without bacteriocin. Bacterial growth was seen to reduce at different concentrations based on the OD of the culture medium recorded at the early hours. Contrary to expectations, dilution of the recombinant peptide was associated with a greater decrease in sample OD (growth reduction), (Pearson coefficient: 0.99; *p* ≤ 0.05 in regression analysis) (Fig. 9).

Our findings indicated recombinant laterosporulin had the greatest inhibitory effect (50.08%) at a concentration of 3.9 µg/ml on the growth of *S. aureus* and its bacteriostatic property was revealed.

## Discussion

*Brevibacillus laterosporus* strains have attracted the attention of researchers because they are non-pathogenic bacterium capable of synthesizing a significant number of antimicrobial molecules. Members of the species *Br. laterosporus* are not pathogenic or harmful to eukaryotic cells and have even been described as probiotics. In recent years, various peptides with broad-spectrum antimicrobial activity have been isolated from strains of *Br. laterosporus* [16].

Laterosporulin 10 is a class IId bacteriocin from strain *Brevibacillus* sp. SKDU10 with a molecular weight of 1–2 kDa [17] and exhibited only 57.6% homology to laterosporulin [18]. Furthermore, this bacteriocin has a different antimicrobial activity spectrum compared to latrosporulin as its inhibitory activity is limited to Gram-positive bacteria [18].

Miljkovic, et al. investigated the antibacterial peptides of three new isolated *Br*. *laterosporus* BGSP7, BGSP9 and BGSP11 strains from silage. The molecular weight of these peptides was 1.5 and 6 kDa and these peptides show inhibitory activity towards a number of pathogenic bacteria [16].

Remarkable properties such as a wide range of antibacterial spectrum, strong effect at low concentration on *S. aureus* bacteria [8] and defined crystal structure of the peptide [9] were the reasons for overexpression of laterosporulin. Previous studies have shown the difficulty of expressing the bacteriocin laterosporulin [16]; Nevertheless, we were able to express this bacteriocin by using the N-SH2 domain.

The production of a soluble recombinant form of laterosporulin is difficult, due to the presence of three disulfide bonds in its structure as well as the toxicity of this peptide to the host. This confirms the need for using a chaperone in the structure of this peptide.

By providing suitable conditions for the oxidation of cysteine residues in proteins, chaperones may prevent the irreversible disruption of protein conformation and play a role in the formation of disulfide bonds and solubility of a protein [19].

According to our bioinformatics data, the N-SH2 domain is a molecule with complete hydrophobic surfaces that can act as a suitable chaperone.

In Fox et al.’s. experiment, the presence of broad hydrophobic surfaces on MBP (Maltose-binding protein) protein was attributed to its chaperone effect that facilitated the proper folding of proteins fused to MBP [20].

Bioinformatics analysis exhibited that the designed structure has a favorable physicochemical structure, and the N-SH2 domain in terms of solubility and proper folding can be helpful in the production of recombinant bacteriocin in vitro.

It has been determined that adding a fusion protein to the N-terminal portion of group IIa of bacteriocins induces an adequate degree of flexibility [21]. Regarding this feature, in our new construct, the sequence of the N-SH2 domain was designed at the N-terminal site of the laterosporulin encoding gene.

Based on the results, the presence of the N-SH2 domain was observed to be favorably effective in expressing the desired refolded protein (105 mg/l). The expression of protein increased markedly in the early hours after the induction of the cell by an exceptionally minute quantity of IPTG (0.01 mM). The expression of the fusion protein was examined at 37, 30, and 16°C. Contrary to the expectation, the expression of the fusion protein did not reflect a decreasing trend at varied temperatures (data not shown). On the other hand, Ahmad and colleagues demonstrated that a high expression level of protein-tyrosine phosphatase was related to the presence of the SH2 domain [22]. Fairlie demonstrated that the engineered mutant of the N-terminal SH2 domain as fusion protein could improve the expression of short peptides [12] which supports our results as well.

In the field of recombinant bacteriocin production, various ranges of production yields, including 26 mg/l of pisicolin [23], 20 mg/l of enterolysin [24], and 23.1 mg/l of divercin 41 [25], have been reported so far. In the current study, after the in vitro folding and cleavage digestion process, 15.75 mg/l of the pure recombinant laterosporulin was achieved. Most importantly, the use of the fusion partner resulted in a high expression-level, easy, and cost-effective folding approach. Although we obtained a considerable expression of the refolded fusion protein (105 mg/l) by using the N-SH2 domain as the partner, this does not imply there is a direct relationship between the carrier protein and the increase in the yield of recombinant peptides. As a matter of fact, the effectiveness of a carrier protein domain may be differently associated with its certain fused AMP [26, 27].

To the best of our knowledge, this is the first experiment to report on the recombinant production of a bacteriocin having three disulfide bonds in its intrinsic conformation.

Laterosporulin comprises 6 cysteine residues, which form three possible disulfide bonds during the folding of the recombinant peptide. Lack of a cysteine residue in the N-SH2 domain [12], leads to the advantage of preventing disulfide bond formation between the partner and target peptide.

Interestingly in our experiment, peptide folding containing three disulfide bonds was achieved in vitro without the necessity for oxidizing and reducing reagents. Although there is no assurance that the folding process is perfectly like the naturally occurring peptide, but the N-SH2 domain showed a desirable potential to act as a chaperone and helped to form the disulfide bonds and a soluble refolded fusion protein. The presence of the N-SH2 partner could enhance the folding with a yield of 77.92% in the dialysis phase.

The results revealed that the N-SH2 domain has a potentially beneficial impact on the folding, solubility, and expression of the recombinant laterosporulin.

During a time-dependent reaction, after 18 h of the enterokinase digestion of fusion protein**,** the presence of 50 mM NaCl in protein solution resulted in producing a soluble and pure laterosporulin. Inferentially, the sedimentation of the fusion protein and N-SH2 partner after digestion could be due to the increase of surface hydrophobicity of the N-SH2 domain and fusion protein, and the high ionic strength in the buffer.

N-SH2 domains are likely to inhibit the formation of accidental hydrophobic interactions by attaching to the hydrophobic surfaces of misfolded or unfolded proteins. As a result, the N-SH2 domain can prevent the irreversible disruption of protein conformation, aggregation and protein precipitation.

Similarly, Bonetti et al. revealed that the electrostatic attraction between Gab2 (GRB2 Associated Binding Protein 2)_608–620_ and N-SH2, was the factor that caused the fast aggregation and formation of the confrontation complex [28]. Other recent findings have exhibited that the SH2 domain consists of a large central hydrophobic antiparallel β-sheet (3 to 5) [29] and acts as a conformational switch [30]. By using the N-SH2 domain’s hydrophobic property, the recombinant peptide purification was easily achieved without the use of additional equipment and costs. Also, the placement of the enterokinase cleavage site as a flexible fragment between the N-SH2 domain and the laterosporulin sequence facilitates peptide rotation and the folding process.

The cleavage of the N-SH2 domain from the fusion protein took place at room temperature. Also, the stability of recombinant laterosporulin solubility at room temperature indicates the formation of disulfide bond and subsequent preservation of the peptide conformation. However, this peptide maintained its stability at 4°C.

Singh et al. examined the Far-UV CD spectrum of laterosporulin peptide both in the disulfide bond formation and non-disulfide bond formation states [9]. They reported an atypical CD spectrum for laterosporulin peptide with the positive band at 228 nm. Probably, this is related to either the presence of extensive disulfide bonds or the appearance of aromatic interactions in this peptide [9]. According to the study by Singh and colleagues, the presence of a positive band at 228 nm in the Far-UV CD spectrum of the recombinant peptide can be a reason for the formation of disulfide bonds. From the results of the Far*-*UV CD spectrums, a comparison of the two structures revealed a similarity between the abundant β-sheets-based structural motif and lower alpha-helix of both the recombinant and natural laterosporulin peptides [9]. However, minor differences in the CD spectrum of the recombinant peptide with the natural form of the peptide may be due to experimental error or the possible presence of some peptides with incomplete disulfide bonds (< 100%).

In the present study, the inhibitory effect of the recombinant peptide on the growth of *S. aureus* began at a concentration of 250 µg/ml and the greatest effect (50.08%) was at a concentration of 3.9 µg/ml. Examination of the antibacterial effect by peptide dilution showed that, an increase in dilution was linked to an increase in growth inhibitory properties contrary to expectations.

It is speculated that the conformation of total peptides is not absolute, and dilution of the peptides increased the ratio of peptides with the correct folding in relation.

According to Baneyx et al., the reduction of bacteriocin activity of the laterosporulin might be due to the oligomerization in the elevated concentrations [31]. A growing body of evidence has demonstrated the decrease in potency of recombinant bacteriocins, such as Sakacin A, Divercin v41, Enterocin A, and Pediocin PA-1, when compared to the wild type samples [32]. For instance, *Clostridium botulinum* 62A transconjugant carrying boticin B induced a smaller growth inhibition zone (2 mm) compared with the wild type *C. botulinum* 6213B (8 mm) in culture filtrate [33]. Consistent with these data, the antibacterial activity of recombinant enterolysin A was revealed to be 8.5 fold lower than its native form, when the protein was overproduced in the *E. coli* system [24]. Accordingly, the wild type of laterosporulin was exhibited to be more active than the recombinant peptide.

By considering the cleavage site for the *Hin*dIII enzyme in the design of the construct, the leucine residue was inevitably placed at the N-terminal portion. The decrease in the bacteriocin activity could be attributed to the presence of additional leucine residue. According to the research conducted by Kazazic, the presence of an additional lysine residue at the N-terminal part of the bacteriocin sakacin*-*P was shown to decrease the bactericidal potency of bacteriocin [21].

## Conclusion

The genetic construct design and the use of various tags provided a considerable advantage to the production of recombinant bacteriocins, resulting in a large mass and potent bacteriocin. The advantages of the use of the N-SH2 domain as a chaperone comprise the augmentation in protein expression, rapid and easy approaches for folding, and purification in a reasonable yield and cost-effective fashion. The N-SH2 domain is promising as a fusion partner not only for the design of bacteriocins due to its low molecular weight of about 15 kDa but also for the overexpression of other protein having disulfide bonds**.**

## Methods

### Bioinformatics tools

Bioinformatics analysis included assessing the effect of the N-SH2 domain on fusion protein solubility, as well as the hydrophobicity of the N-SH2 domain and fusion protein. After which, the effects of the N-SH2 domain on protein folding in vitro was then predicted.

The secondary structure of the N-SH2-LTS fusion protein was predicted by Phyre2 server. Protein-sol patches software was also used to determine the solubility, and to investigate the hydrophobicity and hydrophilicity of the fusion protein components [34, 35].

### Plasmid construction

To design a new construct, the N-terminal fragment of the SH2 domain [12] and the sequences of the laterosporulin gene (153 bp) were retrieved from NCBI (accession number: HE579167). The sequence of the N-SH2 domain was placed on the N-terminal region upstream of the laterosporulin nucleotide fragment, where the PelB and His*-*tag sequences were located. Furthermore, a site for enterokinase digestion was considered between the N-SH2 domain and laterosporulin sequence. We also included restriction enzyme sites for *Nde*I and *Xho*I flanking our construction. Concerning the *E. coli* k12 codon preference, the sequence was optimized, and the 603 bp DNA fragment was ordered to be synthesized by General Biosystems (USA).

### Subcloning and expression of the recombinant laterosporulin

#### Gene cloning

The synthesized fusion gene fragment was first cloned in pET-28a by General Biosystems (USA). To sub-clone, the desired gene fragment into the pET-22b(+) plasmid, the pET28a-N-SH2-LTS and the pET-22b(+) were separately subjected to double digestions by *Nde*I and *Xho*I (Roche, Germany). Afterward, the purified fragment was ligated to the previously digested pET-22b(+) using T4 DNA ligase (Thermo Fisher Scientific, USA) at 16°C overnight. Then, the recombinant pET-22b-N-SH2-LTS plasmid was transformed into the *E. coli* BL21 (DE3) cells provided by Molecular Biotechnology Laboratory (Department of Biology, Faculty of Science, Shiraz University).

Double digestion by the restriction enzymes (*Xho*I and *Nde*I) was used to confirm the gene cloning in pET-22b(+). Besides, to confirm the successful cloning of the insert in the new construct, the recombinant plasmid was sequenced by Med genome Corporation.

### Expression of the fusion protein

Colonies containing the synthetic fragment of 603 bp (carrying PelB, N-SH2 domain, enterokinase cleavage site sequence and LTS) were inoculated into 20 ml of LB broth having 0.1 mg/ml ampicillin and grown overnight at 37°C. These cultures were used to inoculate 1000 ml of LB broth encompassing 0.1 mg/ml ampicillin and grown to an OD_600_ of 1. This was preceded by induction with the final concentration of 0.5 mM IPTG (Cinnagen Company, Iran) at 30°C overnight. The following day, the cultures were centrifuged at 8500 × *g* for 15 min and the supernatant was poured away.

Subsequently, the expression of the fusion protein was monitored by SDS-PAGE analysis (12% polyacrylamide, Sigma) [36]. Western blotting was used to analyze the expression of the fusion protein. The membrane was incubated with a 1:1000 dilution of monoclonal anti-polyhistidine-peroxidase (Sigma, USA). Proteins were visualized using H_2_O_2_/DAB substrate/ chromogen (Sigma, USA).

### Purification, in vitro refolding and digestion of fusion protein

The cells were resuspended in lysis buffer (20 mM Tris–HCl; 300 mM NaCl, pH 8.0) and sonicated for 6 min with 30S intervals (65% frequency) (Bandelin, Germany). After centrifugation, the cell biomass was washed with the lysis buffer twice and centrifuged again to obtain inclusion bodies.

To extract the recombinant fusion protein, inclusion bodies were dissolved in the lysis buffer containing 8 M urea to a 1:20 volume ratio. The mixture was kept at room temperature for half an hour, followed by centrifugation (20 min, 8500 × *g*). The supernatant was poured into the Ni–NTA agarose column (Qiagen, Hilden, Germany) and the His-tagged fusion protein was eluted by gradient concentration of imidazole (5–250 mM). The purified sample was dialyzed against the buffer (20 mM Tris–HCl, pH 8.0), and allowed to refold the fusion protein by incubation at 4°C for 24 h and exchanging the buffer three times.

To evaluate the antimicrobial activity of the peptide, it was necessary to remove the partner protein and His*-*tag from the recombinant peptide. Therefore, 10 mg of the fusion protein was treated with 10 units of enterokinase (Bio Basic, Canada) in the presence of 20 mM Tris–HCl, 50 mM NaCl, and pH 8 for 18 h at room temperature (25°C). The purity of the recombinant laterosporulin was analyzed by reversed-phase high-performance liquid chromatography (RP-HPLC) using a TSK reverse phase C_18_ column (7.8 × 300 mm). It was eluted with ddH_2_O-TFA 0.4% (ElutA) and Acetonitrile (ElutB) in a ratio of 70:30, and a flow rate of 1 ml/min for 20 min. The detection wavelength was 230 nm.

### Tris-Tricine SDS-PAGE

The polyacrylamide gel (T = 15%, C = 5%), as a uniform separating gel and buffers for Tris-Tricine-SDS-PAGE, was prepared according to Schagger and von Jagow [37]. After digestion, protein samples were fixed on the gel and dyed with Coomassie Brilliant Blue R-250 (Merck, Germany).

### Circular Dichroism (CD) measurements

CD spectra were obtained on a Circular Dichroism Spectrometer (Model-215, USA). The spectrum was measured from 190 to 260 nm and scrutinized using a 0.1 cm path length quartz cuvette at 25°C with 200 μg/mL of laterosporulin concentration. Data were obtained at 0.1 nm resolution with a scan rate of 100 nm/min. Then, the prediction of the percentage of the secondary structure of the protein was performed using the CDNN software based on the data obtained from the CD spectrum.

### Antimicrobial test

The antimicrobial activity of the recombinant laterosporulin was investigated against *S. aureus* (ATCC 1556) provided by Microbiology Laboratory (Department of Pathobiology, School of Veterinary Medicine, Shiraz University). The effects of this peptide were estimated in triplicate using a microtiter plate dilution assay. Briefly, *S. aureus* was inoculated into the sterile culture medium from overnight culture, followed by adjusting the turbidity to a 0.5 McFarland standard. Besides, we added 100 μl sterile Brain Heart Infusion (BHI) culture medium to the wells of each row, preceded by 100 µl of the peptide (500 µg/ml) to the first well. This finally produced a 1:2 serial dilution. Additionally, two microliters of culture medium inoculated with *S. aureus* adjusted to the turbidity of a 0.5 McFarland. The microtiter plates were incubated at 32°C in a shaking incubator, and the OD (at 600 nm) was measured at different time intervals by an ELISA plate reader (BioTek MQX200R2, USA).

## Supplementary Information

**Additional file 1: Fig. S1.** The uncropped images of Fig. 4. **Fig. S2.** The uncropped images of Fig. 5. **Fig. S3.** The uncropped images of Fig. 6.

## Data Availability

The sequencing data of the N-SH2-LTS fusion protein is presented on the NCBI database (GenBank accession number MZ496307). The datasets used and/or analyzed during the current study are available from the corresponding author on reasonable request.
