# Supplementary material for: A novel approach of recombinant laterosporulin production using the N-SH2 domain of SHP-2
Source: BMC Biotechnol. 2021 Oct 21;21:60. doi: 10.1186/s12896-021-00721-7 (PMC8529825; doi:10.1186/s12896-021-00721-7)
Supplement: Supplementary file 1 — Additional file 1: Fig. S1. The uncropped images of Fig. 4. Fig. S2. The uncropped images of Fig. 5. Fig. S3. The uncropped images of Fig. 6. [file 12896_2021_721_MOESM1_ESM.docx]

**A novel approach of recombinant laterosporulin production using the N-SH2 domain of SHP-2**

Simin Salehzadeh^1^, Mohammad Tabatabaei^1^*, Abdollah Derakhshandeh^1^, Hamid Reza Karbalaei-Heidari^2^, [Nasrin Kazemipour](https://www.ncbi.nlm.nih.gov/pubmed/?term=Kazemipour%20N%5BAuthor%5D&cauthor=true&cauthor_uid=29362600)^3^

1: Department of Pathobiology, School of Veterinary Medicine, Shiraz University, Shiraz, Iran

2: Department of Biology, Faculty of Science, Shiraz University, Shiraz, Iran

3: Department of Basic Science, School of Veterinary Medicine, Shiraz University, Shiraz, Iran

**Correspondence to:**Mohammad Tabatabaei

**Email:** [Mtabatabaei2003@yahoo.co.uk](mailto:Mtabatabaei2003@yahoo.co.uk)

**Title: The original, unprocessed versions:**

**Figure S1** The uncropped images of figure 4.


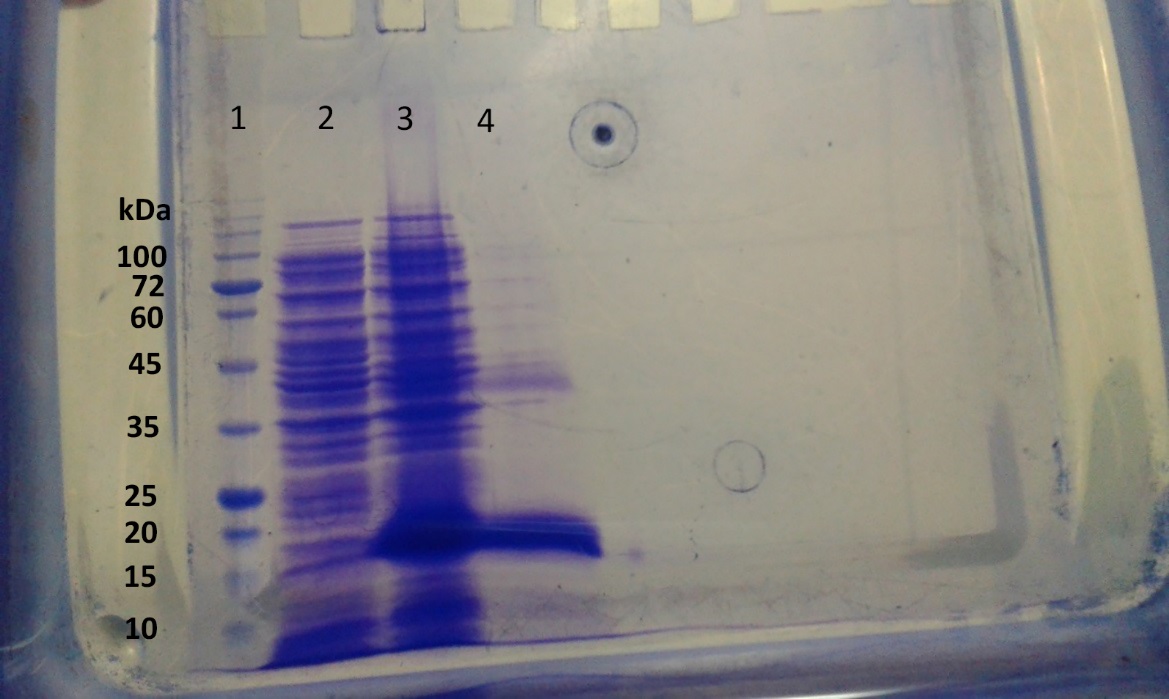


**Figure S1** The uncropped images of figure 4. The presence of a strong expressed protein band was visualized on SDS-gel with a molecular weight of 21.78 kDa in cell lysate. (Lane1): Protein marker, (lane 2): Transformed E. coli cell lysates before induction with IPTG, (lane 3) :Transformed E. coli cell lysates after 18 hrs induction with 0.5 mM IPTG, where the presence ofa strong expressive protein is shown at a weight of 21.78 kDa , ( lane 4): Purified denatured fusion protein

**Figure S2** The uncropped images of figure 5.


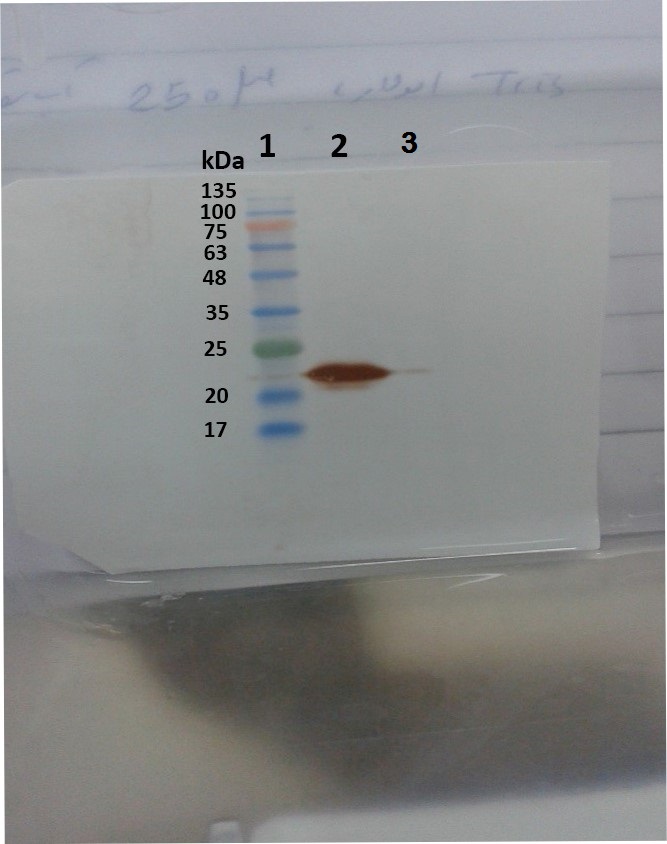


**Figure S2** The uncropped images of figure 5. Western blot analysis of E. coli cells lysates in order to confirm the molecular weight of expressed fusion protein . (Lane 1): Protein ladder, (Lane 2): Fusion protein was visualized at a weight of 21.78 kDa, (Lane 3): cells lysates without inductionwith IPTG

**Figure S3** The uncropped images of figure 6.


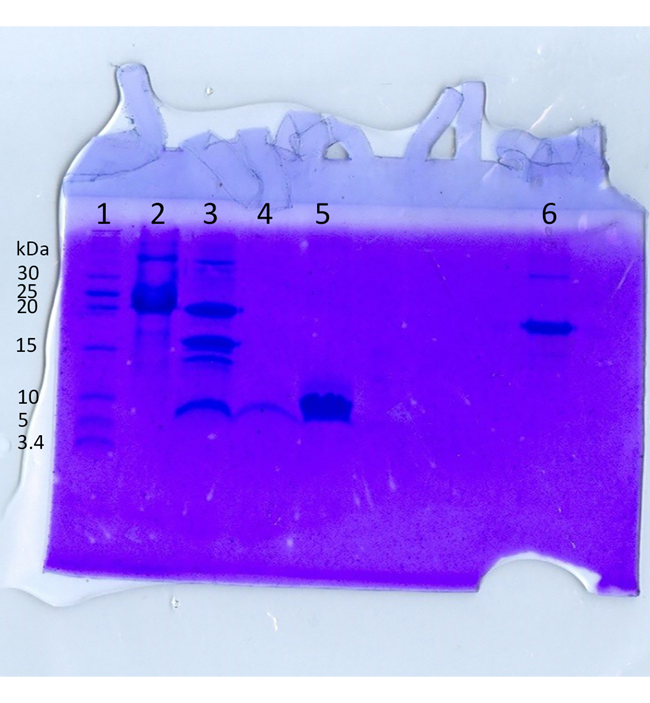


**Figure S3** The uncropped images of figure 6. Tris-Tricine SDS-PAGE (15% polyacrylamide gel) profiles of fusion laterosporulinbefore and afterenterokinase digestion.(Lane 1): Protein ladder, (Lane2): Undigested refolded fusion protein at a weight of 21.78 kDa,(Lane3): Digested fusion protein with enterokinase,(Lane 4): The purified recombinant laterosporulin (supernatant) at a weight of 5.75 kDa, (Lane 5): Highly concentrated laterosporulin, (Lane 6):Another sample of undigested refolded fusion protein at a weight of 21.78 kDa.
